# Severe Diabetic Ketoacidosis in Children with Type 1 Diabetes: Ongoing Challenges in Care

**DOI:** 10.3390/children12010110

**Published:** 2025-01-19

**Authors:** Simone Foti Randazzese, Mariarosaria La Rocca, Bruno Bombaci, Alessandra Di Pisa, Elèna Giliberto, Teresa Inturri, Daniel Militi, Fortunato Lombardo, Eloisa Gitto, Giuseppina Salzano, Stefano Passanisi

**Affiliations:** 1 Department of Human Pathology in Adult and Developmental Age “G. Barresi”, University of Messina, 98122 Messina, Italy; simone.fotirandazzese@studenti.unime.it (S.F.R.); mariarosaria.larocca@studenti.unime.it (M.L.R.); brunobombaci@gmail.com (B.B.); alessandra.dipisa@studenti.unime.it (A.D.P.); elena.giliberto@studenti.unime.it (E.G.); teresa.inturri@studenti.unime.it (T.I.); daniel.militi@studenti.unime.it (D.M.); fortunato.lombardo@unime.it (F.L.); gsalzano@unime.it (G.S.); 2 Department of Clinical and Experimental Medicine, Neonatal and Pediatric Intensive Care Unit, University of Messina, 98122 Messina, Italy; egitto@unime.it

**Keywords:** complications, incidence, intensive care unit, pediatrics, prevention, risk factors

## Abstract

Diabetic ketoacidosis is the most common acute complication in children and adolescents with type 1 diabetes, and contributes significantly to morbidity, mortality, and healthcare burden. This review aims to explore the multifaceted aspects of severe diabetic ketoacidosis in pediatric age, including its epidemiology, pathogenesis, risk factors, complications and emphasizing advances in prevention strategies. Incidence rates vary due to influences from geographic, socioeconomic, cultural and demographic factors. Pathogenesis is linked to insulin deficiency and an excess of counter-regulatory hormones, which disrupt glucose, protein, and lipid metabolism, causing hyperglycemia, ketosis, acidosis, dehydration, and electrolyte imbalances. According to the International Society for Pediatric and Adolescent Diabetes guidelines, severe diabetic ketoacidosis is characterized by a pH < 7.1 or bicarbonate < 5 mmol/L. This condition can lead to a wide range of life-threatening complications, including cerebral edema that represents the leading cause of death. Several prevention strategies, including awareness campaigns, early diagnosis of diabetes, regular monitoring and management, effective insulin therapy, education, access to healthcare and technological assistance, may contribute to reduce the risk of severe diabetic ketoacidosis episodes in children and adolescents.

## 1. Introduction

Diabetic ketoacidosis (DKA) is a severe and potentially life-threatening metabolic complication of diabetes that can occur in people with diabetes [1]. This condition presents unique challenges for healthcare providers, particularly in children, where it represents a leading concern in diabetes management [2].

DKA is defined by the following clinical and biochemical alterations: hyperglycemia (blood glucose > 11 mmol/L or approximately 200 mg/dL), metabolic acidosis (venous pH < 7.3 or serum bicarbonate < 18 mmol/L), and ketonemia (blood β-hydroxybutyrate ≥ 3 mmol/L) or moderate to large ketonuria [3]. These abnormalities arise from an absolute or relative deficiency of insulin associated with an excess of counter-regulatory hormones, leading to significant disruptions in glucose, protein, and lipid metabolism [4]. Glycogen stores are rapidly depleted, triglycerides in adipose tissue undergo hydrolysis, and amino acids are mobilized from muscle tissue [5]. These substrates are transported to the liver, where they serve as precursors for the synthesis of glucose and ketone bodies [5]. This metabolic cascade results in severe hyperglycemia and accumulation of ketones, which are key drivers of the metabolic dysregulation observed in DKA [6]. The resulting acidosis, combined with dehydration and electrolyte disturbances, underscores the complexity and urgency of managing this condition [6].

DKA is the most common hyperglycemic emergency and a leading cause of mortality in youth with type 1 diabetes (T1D) [7]. It commonly represents the initial clinical presentation of T1D in children [8]. However, DKA may also occur in individuals with previously diagnosed diabetes as the result of insulin omission, either inadvertent or deliberate [9]. Contributing factors such as socioeconomic disparities, limited access to healthcare, and inadequate diabetes education exacerbate the risk of severe DKA, particularly in vulnerable populations [10].

The International Society for Pediatric and Adolescent Diabetes (ISPAD) has established criteria for classifying the severity of DKA based on the degree of acidosis. Mild DKA is characterized by a venous pH of <7.3 or serum bicarbonate < 18 mmol/L, moderate DKA is defined by a pH < 7.2 or bicarbonate < 10 mmol/L, while severe DKA is indicated by a pH < 7.1 or bicarbonate < 5 mmol/L [3]. These classifications are critical for guiding treatment strategies and predicting potential complications.

Despite advances in diabetes care and management, episodes of severe DKA are still associated with significant morbidity and mortality, emphasizing the importance of early recognition and prompt intervention [11]. Challenges such as delayed diagnosis, comorbid conditions, and variability in clinical presentations can complicate the management and increase the risk of adverse outcomes [12]. Enhanced efforts in diabetes prevention and education, improved access to care, and addressing socioeconomic disparities are essential in reducing the incidence and severity of DKA in children and adolescents [10].

This review aims to provide a comprehensive overview of severe DKA in children and adolescents, focusing on epidemiology, pathogenesis, risk factors, clinical presentation, complications and preventing strategies. Relevant articles were identified through a systematic search conducted using the PubMed database. Key terms such as “severe diabetic ketoacidosis” OR “severe DKA” AND “epidemiology” AND “physiopathology” AND “risk factors” AND “complications” AND “prevention” AND “pediatric age” OR “pediatrics” OR “children” were included in the search. Reviews, systematic reviews, meta-analyses, observational studies, randomized controlled trials (RCTs), and evidence-based guidelines of the major scientific societies published between 1999 and 2025 were included to capture the latest evidence and clinical insights. Additional sources were identified through the references of selected articles. Data were synthesized thematically. No formal meta-analysis was performed, given the narrative nature of the review.

## 2. Epidemiology

DKA is particularly prevalent among children and adolescents with T1D, contributing to increased hospitalizations, intensive care, recurrence, and mortality [13]. Although less common, adolescents with type 2 diabetes (T2D) may also present with DKA, particularly those of African or Hispanic descent [14,15]. Specifically, according to the American Diabetes Association (ADA), DKA occurs in 11% of subjects aged 10–19 years at the onset of T2D [16].

According to the ISPAD, DKA frequently occurs at the onset of T1D, with reported prevalence rates ranging from approximately 15% to 70% across Europe and North America. Although incidence rates vary worldwide, several countries have observed a recent rise in the frequency of DKA at the time of T1D diagnosis [3,17,18]. Unfortunately, national registries are not universally available. Therefore, most epidemiological data on pediatric DKA are mainly derived from hospital discharge records.

The frequency of DKA at diagnosis is inversely correlated with the prevalence of T1D in the general population, suggesting that living in regions with higher T1D may allow an earlier recognition of new-onset symptoms, thereby preventing progression to DKA [19,20,21].

A longitudinal population-based study revealed a DKA rate of 40.3% at the time of T1D diagnosis in Italian children <15 years from 2004 to 2013. Among DKA cases, 29.1% were classified as mild/moderate and 11.2% as severe. Younger children and those living in Southern Italy were reported to have a higher risk for severe DKA compared to children from other regions of the country. Conversely, family history of T1D and residence in Sardinia were significantly associated with a reduced likelihood of developing severe DKA [22]. Similarly, a hospital-based cross-sectional study targeting 395 children under 12 years of age diagnosed with DKA and admitted to pediatric wards of Addis Abeba (Ethiopia) between January 2009 and December 2014 documented that children aged 9–12 years and those with parents unaware of DKA signs and symptoms were at particularly high risk of developing severe DKA [23]. Recent data from the German-Austrian Diabetes Registry showed (Diabetes-Patienten-Verlaufsdokumentation—DPV) showed that among 41,189 children and adolescents with newly diagnosed T1D between 2000 and 2019, 19.8% experienced DKA, with a slight increase observed over the study period. The overall severe DKA rate was 6.1%. DKA was more frequent in children under 6 years (21.7%) compared to adolescents aged 12–17 years (18.6%). Furthermore, girls had a higher incidence of DKA than boys (20.5% versus 19.2%) and patients with family history of migration were more likely to present with DKA (21.4% versus 18.2%) [24].

Multiple studies have reported an increase in DKA and severe DKA cases at diabetes diagnosis during the COVID-19 pandemic. Rapid changes in healthcare systems and public behavior likely contributed to this rise [25,26,27]. A systematic review and meta-analysis reported that during the first year of the pandemic, in 2020, new-onset T1D cases rose by 9.5%, DKA by 25%, and severe DKA by 19.5% compared with pre-COVID-19 pandemic [28]. Similarly, Meregildo-Rodriguez et al. conducted a systematic review of 46 observational studies up to 31 August 2022, involving 159,505 children and 17,547 DKA events. The findings showed that the pandemic increased the odds of DKA and severe DKA in children with T1D by 68% and 84%, respectively. Additionally, the odds of DKA were 75% higher among children with newly diagnosed T1D compared with the pre-COVID-19 era [29]. Cherubini et al. evaluated the prevalence of DKA at the onset of T1D in Italy during the COVID-19 pandemic in 2020 and compare it to the prevalence observed between 2017 and 2019. The overall prevalence of DKA rose from 35.7% in 2017–2019 to 39.6% in 2020. Similarly, severe DKA prevalence significantly increased from 10.4% to 14.2%. This trend was more evident during the early phase of the pandemic. Children from immigrant families were more likely to present with DKA (*p* < 0.001), whereas those from households with higher income levels had a reduced likelihood (*p* = 0.010) [30]. In a retrospective study from Iran, the prevalence of DKA cases among children with new-onset T1D during the COVID-19 pandemic (44.7%) was remarkably higher than in the pre-pandemic period (12.6%). Furthermore, during the pandemic, 35.7% of DKA cases were severe (versus 21.2% pre-pandemic) [31].

Severe DKA cases, characterized by altered level of consciousness and/or impaired circulation, often require admission to the pediatric intensive care unit (PICU). A single-center retrospective study identified risk factors related to severe DKA, represented by younger age and electrolyte imbalances. Additionally, severe DKA was linked to lower Glasgow Coma Scale (GCS) scores, prolonged PICU stays and recovery times [32].

Another retrospective study analyzed the prevalence and clinical characteristics of severe DKA cases treated in the PICU of a single center over five years (2017–2022). Of 103 children newly diagnosed with T1D, 51.5% met the clinical criteria for DKA, and 18.4% experienced severe DKA, with 9.7% (10 patients) requiring PICU care. Children admitted to the PICU for severe DKA often came from low-income or immigrant backgrounds, and nearly half of them were younger than five years [33]. Similarly, another retrospective study analyzed the trends and clinical features of DKA in children treated in the PICU at the Clinical Hospital Centre Rijeka, Croatia, from 2011 to 2020. Among 194 children diagnosed with T1D during the study period, 24.7% (48/194) presented with DKA at initial diagnosis, indicating a rising trend, particularly in 2020. Of 58 children treated in the PICU for DKA, 48 had newly diagnosed T1D, and 10 were previously diagnosed. Moderate to severe dehydration was observed in 76% of cases at admission [34]. Nigrovic et al. analyzed 1679 DKA episodes in 1553 children under 18 years. Nearly half (47.5%) of DKA episodes occurred in children with newly diagnosed diabetes, and 23.6% of episodes were classified as severe. Children under 6 years with newly diagnosed T1D were not more likely to have severe DKA. Low socioeconomic status was associated with more severe DKA in newly diagnosed children and with recurrent DKA in previously diagnosed children [35].

## 3. Pathogenesis and Risk Factors for Severe DKA

DKA arises from the progressive β-cell dysfunction in subjects with previously undiagnosed diabetes or from insulin omission, pump failure, or inadequate insulin administration in established diabetes during infections, surgery, trauma, or stress [36]. In these conditions, insufficient insulin leads to uncontrolled hepatic glucose production by the liver and kidneys, impaired peripheral glucose uptake, and enhanced lipolysis, contributing to ketogenesis [37]. This insulin deficiency mimics the pathophysiological mechanisms of hypoglycemia, triggering the release of counter-regulatory hormones (e.g., cortisol, glucagon, catecholamines, and growth hormone), which elevate serum glucose levels by reducing peripheral glucose use, promoting hepatic gluconeogenesis, and stimulating glycogenolysis [38,39,40]. Additionally, hormone-sensitive lipase activity in adipose tissue increases, breaking down triglycerides into glycerol and elevating free fatty acid levels, converted into ketones, such as acetoacetate and β-hydroxybutyrate, through hepatic ketogenesis [40,41]. Among these, β-hydroxybutyrate predominates and contributes significantly to the development of metabolic acidosis [37,38,39]. Hyperglycemia also induces osmotic diuresis, leading to severe dehydration and electrolyte imbalances (e.g., hyponatremia, hypokalemia, hyperkalemia, hyperchloremia, hypophosphatemia, hypocalcemia and hypomagnesemia) [40]. Both hyperglycemia and elevated ketones levels worsen osmotic diuresis, resulting in hypovolemia and a reduced glomerular filtration rate, which further intensifies hyperglycemia [41]. Furthermore, hyperglycemia, combined with ketoacidosis, triggers proinflammatory cytokines release and oxidative stress, further impairing insulin secretion and sensitivity [42]. Finally, free fatty acids may inhibit nitric oxide production, leading to endothelial dysfunction [43].

**Figure 1:**
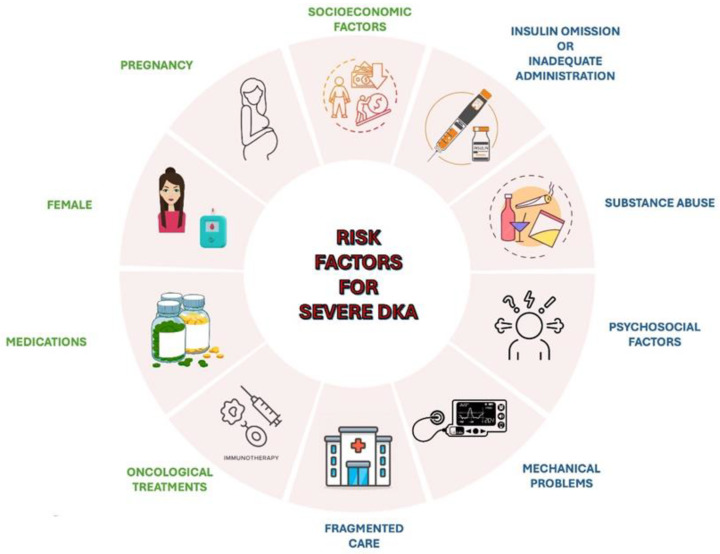
An overview of the main risk factors for severe DKA.

Different risk factors may contribute to the occurrence of severe DKA (Figure 1) [44,45]. These include drugs affecting carbohydrate metabolism, such as thiazides, corticosteroids, sympathomimetics (e.g., dobutamine, terbutaline), and atypical antipsychotics, which may precipitate DKA in susceptible individuals [46]. Socioeconomic factors, including low income, area-level deprivation, homelessness, and lack of health insurance, are strongly associated with increased DKA risk [47,48]. Other contributors include physiological conditions like pregnancy, where DKA may develop at lower glucose concentrations and present with nonspecific symptoms, such as nausea and vomiting [49]. Females have a higher risk for DKA-related hospital admissions and recurrent episodes, potentially due to suboptimal glucose control, body image concerns, and higher prevalence of mental health issues [50]. Additionally, oncological treatments, such as immune checkpoint inhibitors used in cancer therapy, have been linked to an increased risk of DKA [51].

Recurrent DKA episodes are often associated with specific factors. Adolescents are particularly vulnerable due to psychosocial factors, such as intentional insulin omission, which may stem from rebellion or the psychological challenges of chronic disease management, or inadequate administration, especially during periods of increased stress, such as infection, surgery, trauma, or illness, when insulin requirements typically rise [5,50,52]. Mechanical problems with continuous subcutaneous insulin infusion (CSII) may also precipitate DKA [53]. Substance abuse, especially cocaine, alcohol and cannabis, represents a significant risk factor for recurrent DKA episodes [54,55]. Persistent socioeconomic barriers and fragmented care, characterized by recurrent admissions for DKA at multiple hospitals, significantly increases the likelihood of multiple DKA episodes [56].

## 4. Severe DKA-Related Complications

Severe DKA can lead to a wide range of complications, from metabolic and electrolyte alterations to life-threatening systemic conditions, which can lead to multiple organ failure (MOF) and finally result in death if they are not recognized and promptly managed [57,58].

In developed countries, the overall mortality rate for children with DKA is relatively low, ranging from approximately 0.15% to 0.3% [59]. However, when complications occur, the mortality rate increases significantly. Children with DKA and cerebral edema (CE) have a mortality rate of 6.4%, compared to 0.1% in those without CE [60].

**Figure 2:**
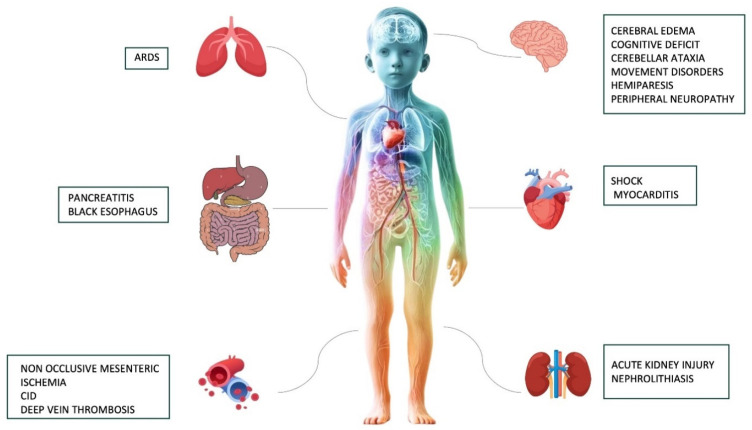
A summary of the most common short and long-term complications associated with severe DKA.

An overview of the major short and long-term complications associated with severe DKA is provided in Figure 2.

### 4.1. Short-Term Complications

#### 4.1.1. Cerebral Edema

CE is one of the most serious complications associated with severe DKA and represents the leading cause of DKA-related morbidity and mortality [61]. It occurs in approximately 0.3–1% of cases, typically manifesting within 4–12 h after the initiation of therapy, although delayed presentations beyond this window are occasionally reported. The condition is associated with a high fatality rate, ranging from 20% to 30% [62].

Despite extensive research, the pathogenesis of CE remains not completely known. Current hypotheses suggest that the concomitance of rapid osmotic shifts during fluid resuscitation, alterations in cerebral blood flow, and inflammatory mediators may contribute to the development of CE. In addition, hyperventilation-induced hypocapnia, typical of Kussmaul breathing pattern, may cause cerebral vasoconstriction and decreased cerebral blood flow [63].

Electrolyte imbalances heighten the risk of developing CE and affect its severity. Specifically, severe hyponatremia (Na^+^ < 120 mmol/L) exacerbates CE by promoting osmotic water influx into the neurons, driven by the need to equalize extracellular and intracellular osmotic gradients. Rapid correction of sodium levels may induce additional osmotic stress, further compromising the integrity of neuronal cells and increasing the risk of cellular injury. Hypokalemia (K^+^ < 3.5 mmol/L or lower) disrupts transmembrane ionic gradients essential for maintaining neuronal excitability and osmotic balance, thereby impairing neuronal function and contributing to an increased risk of CE. Hyperchloremia (Cl^−^ > 110 mmol/L) resulting from the administration of chloride-rich fluids can exacerbate metabolic acidosis contributing to osmolality imbalances, thereby potentially worsening CE. Severe hypophosphatemia (PO_4_^3−^ < 0.32 mmol/L) reduces ATP production, compromising neuronal ionic homeostasis. Finally, rapid pH shifts from bicarbonate therapy also worsen intracellular osmolality changes, increasing CE risk [3,40].

Signs and symptoms of CE include headache, systemic hypertension, slowing or irregular heart rate, irregular respiratory patterns, pupillary abnormalities (e.g., unequal or fixed dilation), change in neurologic status (e.g., restlessness, irritability, increased drowsiness, incontinence inappropriate for the patient’s age), cranial nerve palsies, or other specific neurologic signs [64].

A single diagnostic criterion, two major criteria, or a combination of one major and two minor criteria demonstrate a sensitivity of 92% and a false positive rate of only 4% for identifying CE. The diagnostic indicators encompass abnormal motor or verbal responses to painful stimuli, decorticate or decerebrate posture, cranial nerve dysfunction, and abnormal neurogenic respiratory patterns (e.g., Kussmaul breathing). Major criteria include altered mental status, inappropriate incontinence for age, and a sustained reduction in heart rate by 20 or more beats per minute, which is not explained by improved hydration or sleep. Minor criteria comprise vomiting, headache, lethargy, diastolic blood pressure exceeding 90 mmHg, and age below 5 years [3].

Radiological evidence, often observed through computed tomography (CT), can further confirm the presence of CE, although its absence does not exclude the condition [65].

The risk of developing CE is related to certain predisposing factors, including preschool age, severe acidosis, elevated blood urea nitrogen levels, hypocapnia, and improper fluid resuscitation [66]. The identification of severe clinical signs, such as altered levels of consciousness or hemodynamic instability, should alert clinicians about the need to admission to PICU for close monitoring and treatment [67].

#### 4.1.2. Acute Kidney Injury

Children and adolescents presenting with severe DKA experience significant circulatory volume depletion, which contributes to the development of acute kidney injury (AKI) in approximately 30–64% of cases [68]. AKI diagnosis in this population is typically guided by the pediatric Risk, Injury, Failure, Loss, End-stage renal disease (pRIFLE) criteria, which provide a structured framework for assessing renal impairment [69].

The initial phase of AKI is commonly pre-renal, characterized by reduced renal perfusion that is usually responsive to adequate fluid resuscitation. However, in some cases, the condition progresses to intrinsic renal failure, which can be further complicated by acute tubular necrosis and rhabdomyolysis, a rare but harmful cause of renal injury [70].

A strong correlation between the severity of acidosis and the progression of severe AKI has been demonstrated. Severe acidosis, compounded by pronounced dehydration, contributes to a vicious cycle that exacerbates both renal dysfunction and overall metabolic derangement [71].

An uncommon but remarkable risk factor for AKI in severe pediatric DKA is nephrolithiasis. The formation of stones in children is likely driven by a combination of factors, including severe dehydration, persistent acidosis, and glucosuria-induced hypercalciuria [72].

#### 4.1.3. Acute Respiratory Distress Syndrome

Acute respiratory distress syndrome (ARDS) is a rare but serious complication of severe pediatric DKA, which results in a sudden onset of dyspnea and progressive hypoxia [73]. The exact pathogenesis is not fully understood. Proposed mechanisms suggest that severe metabolic acidosis associated with DKA, combined with increased production of pro-inflammatory cytokines (e.g., TNF-α, IL-6) driven by prolonged hyperglycemia, leads to endothelial damage, heightened capillary membrane permeability and fluid accumulation in the alveoli, culminating in pulmonary edema [74]. Timely identification of ARDS can enhance survival rates and yield highly favorable outcomes, as patients with DKA who develop unresponsive ARDS can be effectively treated with Extracorporeal Membrane Oxygenation (ECMO) support [75].

#### 4.1.4. Shock and Myocarditis

Severe dehydration can also lead to circulatory shock, characterized by typical clinical signs such as tachycardia, prolonged capillary refill time (>3 s), cold and clammy extremities, and hypotension, reflecting significant intravascular volume depletion and impaired perfusion [57]. In cases where shock is accompanied by additional cardiological findings, such as elevated troponin I levels, low-voltage QRS complexes on ECG, and a reduced ejection fraction, the possibility of myocarditis should be seriously considered. In this setting, myocarditis may arise secondary to severe metabolic derangements, hypoperfusion, or as part of a broader inflammatory or infectious process. This condition can exacerbate hemodynamic instability and requires prompt recognition and management, including supportive care and potential consultation with a pediatric cardiologist [76].

#### 4.1.5. Deep Vein Thrombosis

Subjects with a genetic predisposition to thrombosis are particularly vulnerable to develop deep vein thrombosis (DVT) due to inflammation, dehydration and hyper-viscosity combined with the disruption of the normal coagulation cascade [77].

In pediatric cases, the risk of DVT is further elevated in children who undergo central venous catheter placement, especially those aged below 3 years. This increased risk may be attributed to the smaller vessel diameter in younger children and the severity of DKA typical of this age [78].

#### 4.1.6. Gastrointestinal Complications

Although rare, gastrointestinal involvement can occur in cases of severe DKA, potentially leading to serious outcomes. Non-occlusive mesenteric ischemia, caused by hypoperfusion and microangiopathy, is the most common gastrointestinal complication among children and adolescents. This condition may present as persistent abdominal pain and severe dehydration, warranting prompt evaluation and intervention [79].

While acute pancreatitis is an uncommon complication, asymptomatic elevation of pancreatic enzymes is frequently observed in youth with DKA. This finding requires careful differentiation from clinically significant pancreatic involvement [80].

Rarely, DKA may lead to upper gastrointestinal bleeding, which can be associated with severe metabolic impairment and stress-related mucosal injury [81]. This complication is usually related to the occurrence of acute esophageal necrosis, also known as “black esophagus” [82,83].

### 4.2. Long-Term Complications

#### Cognitive and Neurological Implications

Severe DKA can result in cognitive impairments and changes in brain development in young children, especially those under five years of age [84]. Even without overt neurological symptoms, these children are at higher risk of cognitive deficits, including impairments in memory, attention, and intelligence quotient (IQ). Indeed, studies grouping children by DKA severity have consistently shown that those with moderate-to-severe DKA exhibit significantly lower full-scale IQ scores and cognitive performance than those with mild or no DKA. Recently, a prospective study investigated whether a single episode of DKA is linked to cognitive declines in children with newly diagnosed T1D and whether similar effects are observed in children with prior diagnoses. Among 758 children aged 6–18 years who experienced DKA, neurocognitive assessment was evaluated 2–6 months after the DKA episode. A comparison group of 376 children with T1D, but no DKA exposure, was also enrolled. Among all subjects, moderate/severe DKA was associated with lower IQ, reduced item-color recall, and shorter forward digit span. For newly diagnosed children, moderate/severe DKA correlated with lower item-color recall. In previously diagnosed children, repeated DKA exposure and higher glycated hemoglobin (HbA1c) levels were independently linked to significant IQ reductions. These findings suggest that while a single DKA episode soon after T1D diagnosis causes mild cognitive effects, repeated episodes and chronic hyperglycemia in previously diagnosed children lead to more substantial cognitive impairments [85].

While severe cerebral injury is rare (<1%), mild cerebral injury is more common and may lead to subtle long-term deficits. Rare neurological complications may include peripheral neuropathy, often associated with other complications such as cerebral injury or disseminated intravascular coagulation [58]. Isolated case reports described conditions such as cerebellar ataxia, movement disorders (e.g., choreiform movements or pill-rolling tremors), and hemiparesis in children [86].

## 5. Prevention Strategies

### 5.1. Primary Prevention Strategies

Raising professional and public awareness about the early signs and symptoms of hyperglycemia is paramount to prevent severe DKA and its associated complications. [87].

Studies have shown that children who are diagnosed with T1D have been visited by a physician several times in the days or weeks before their diagnosis and this pattern is especially common among very young children [37,88].

#### 5.1.1. Awareness Campaigns

During recent decades, various campaigns have been launched to promote education and prevention. An 8-year study (1991–1999) conducted in the province of Parma, Italy, demonstrated that an awareness campaign, named “the Parma campaign”, significantly reduced both the incidence and severity of DKA at diagnosis. This campaign consisted of posters containing practical messages about T1D, which were disseminated to primary and secondary schools and in physicians’ outpatient rooms. Additionally, a dedicated section on the early diagnosis of T1D was added to the mobile application “Kids and Teens Diabetes”, a multilingual reference tool, available in 15 languages, offering guidance for managing diabetes in several contexts, including schools, workplaces, physical activities, travel, and social events. The cumulative frequency of DKA at the onset of T1D dropped from 78% before the campaign to 12.5% afterward [89]. Additionally, the authors showed that the positive effects of the campaign persisted for several years, with no cases of severe DKA reported and a significant decline in mild or moderate DKA [90]. A similar campaign was conducted in Australia collecting data on children diagnosed with T1D in Gosford, Newcastle, and Sydney. Diabetes education, delivered only in Gosford, was inspired by the Parma campaign and used various tools, including explanatory letters, posters, and postcards. The results showed a 64% reduction in the rate of DKA at initial diagnosis and lower blood glucose levels at first presentation during the intervention period compared to the baseline [91].

A recent systematic review showed that the reduction in DKA rates due to awareness campaigns ranges from 1% to 65.5%. This wide range is closely related to the intrinsic characteristics of each campaign. However, a reduction in acute complications (e.g., CE), lower HbA1c levels at diagnosis, increased C-peptide levels, and shorter hospital stays were found. Noteworthy, campaign costs were reported to be significantly lower than the expenses of managing DKA cases [92].

#### 5.1.2. Screening Strategies

In recent years, screening for T1D has been extensively discussed, aiming to the identification of early stages of T1D, reducing the incidence of severe DKA, its associated morbidity, the economic burden of hospitalization, and the psychological impact on families [93]. In 2015, Hummel et al. launched in Bavaria (Germany) the Fr1da study, a project to evaluate population-based screening for multiple islet autoantibodies in children for the early detection of T1D. The study revealed that children diagnosed in early stages exhibited milder symptoms upon progression to overt diabetes than those without prior screening. These children also presented lower HbA1c, lower fasting blood glucose levels, and higher fasting C-peptide levels. Authors also reported that previously screened children occasionally experienced severe DKA (2.5%) and required lower insulin doses at stage 3 of T1D diagnosis [94]. The ASK study, started in 2017 in Colorado, demonstrated that presymptomatic diabetes screening could be cost-effective in regions with high DKA prevalence, reducing DKA rates at diagnosis and improving HbA1c levels [95].

Recently, Italy became the first country to approve a law mandating autoantibody screening for T1D and celiac disease (CD) for all the children and adolescents. As part of this initiative, the “D1Ce Screen” pilot study was launched, targeting 5363 children from four regions (Lombardy, Marche, Campania, and Sardinia). Children are screened in three age groups, 2–2.9 years, 6–6.9 years, and 10–10.9 years, corresponding to the peaks of islet autoantibody seroconversion. In this program, family pediatricians play a central role in identifying children who test positive for islet autoantibodies and referring them to T1D Regional Care Teams for monitoring. Discussions are ongoing to establish effective follow-up protocols for children testing positive, ensuring that post-screening monitoring is both acceptable and efficient for families. Based on the pilot study’s results in 2024, the program is planned to expand to all the Italian regions by 2025 [96].

Meanwhile, some screening programs have been focusing on newborns, assessing human leukocyte antigens (HLA) typing due to the significant genetic risk of T1D associated with HLA DR/DQ alleles [97].

Other programs target genetically at-risk populations to prevent acute complications at initial presentation. Among these, the Diabetes Autoimmunity Study in the Young (DAISY) demonstrated that early detection of islet cell autoantibodies combined with periodic HbA1c testing in genetically at-risk individuals significantly reduced the incidence of hospitalizations at diabetes onset [98].

#### 5.1.3. The Role of Disease-Modifying Therapies

Individuals at early stages of T1D may benefit from disease-modifying therapies [99].

Teplizumab is the first drug approved by the Food and Drug Administration (FDA) in November 2022 for delaying the clinical onset of T1D in subjects aged ≥ 8 years with stage 2 of T1D [100]. This humanized recombinant monoclonal antibody binds to the CD3 chain of T lymphocytes and helps to preserve β-cell and regulatory T-cell function, maintaining insulin secretion rates [101,102]. As a result, stage 3 of T1D may be delayed for an average of approximately 2.7 years [100]. Side effects include lymphopenia, rash, headache, Epstein–Barr virus reactivation, anaphylaxis, angioedema, and urticaria. The drug can also result in transient immunosuppression [101].

A recent study, exploring parents’ perspectives of families about this innovative drug, reported a relative low knowledge and trust in this medication [103]. Despite its potential benefits, teplizumab is limited to a narrowly defined population due to the lack of widespread screening programs for early-stage diabetes outside of research settings. Additionally, the high cost of treatment remains a significant barrier [101,102].

Recently, the FDA has granted Fast Track designation for Diamyd^®^ (rhGAD65/alum; Stockholm, Sweden) to treat T1D in pediatric patients with stage 1 or stage 2 carrying the genotype HLA DR3-DQ2 [104]. Diamyd^®^ is an investigational immunotherapy, based on recombinant human glutamic acid decarboxylase (rhGAD65) formulated with aluminum hydroxide (alum) as an adjuvant, which aims to modulate the immune response by inducing tolerance to GAD65, thereby reducing autoimmune destruction of beta cells. This approach seeks to preserve endogenous insulin production, which could improve blood glucose control and reduce the need for exogenous insulin. Research is ongoing to optimize its efficacy and identify the patient populations most likely to benefit [105].

### 5.2. Secondary Prevention Strategies

Secondary DKA is often triggered by inadequate management of intercurrent illness or stress, or by the accidental or intentional omission of insulin, so prevention strategies are essential [106].

#### 5.2.1. Sick-Day Management and Prevention

Education plays a crucial role in raising awareness about the risks of secondary DKA and training patients and their families to manage effectively sick days. Thus, dedicated protocols should be regularly discussed with youths and their families, especially during winter months when the incidence of acute episodes of illness increases [107].

#### 5.2.2. The Role of Maintaining Optimal Glucose Control

HbA1c reflects long-term blood glucose control and is a critical predictor of diabetes-related complications [108]. While HbA1c has been linked to recurrent DKA, its relationship with DKA severity is less explored. A recent study by Lamsal et al. analyzed 88 children and young adults admitted to PICU for DKA between 2011 and 2015. Mean HbA1c for mild, moderate, and severe DKA groups were 11.4%, 12.2%, and 14.8%, respectively. A positive correlation was found between HbA1c levels and the severity of DKA, emphasizing the need for effective blood glucose control to reduce severe DKA events [109].

#### 5.2.3. Innovative Technologies

In recent decades, several advanced technologies, represented by continuous glucose monitoring (CGM), insulin pumps, and automated insulin delivery (AID) systems, have been implemented to improve the management of T1D, with the dual goals of achieving optimal glycemic control and reducing the incidence of acute complications such as secondary severe DKA and severe hypoglycemia [110,111]. Karges et al. conducted a population-based cohort study involving 446 diabetes centers across Germany, Austria, and Luxembourg as part of the Diabetes Prospective Follow-up Initiative comparing different treatment strategies among adolescents and young adults. Authors reported that insulin pump therapy was associated with lower risks of severe DKA as well as hypoglycemia [112]. The Control-IQ Observational (CLIO) Prospective Study, conducted between August 2020 and March 2022 in 3157 participants from the United States, found lower adverse event rates, including DKA, among individuals using the t-slim X2™ insulin pump with Control-IQ technology [113].

A systematic review and meta-analysis of randomized controlled trials comparing AID to conventional treatment revealed that the rate of episodes of DKA did not significantly differ between groups. However, secondary DKA in pump users was more frequently observed in adolescents with elevated HbA1c levels [114].

#### 5.2.4. Role of Ketone Monitoring

According to the ISPAD guidelines, ketone monitoring plays a critical role in preventing and detecting secondary DKA, especially during any acute illnesses, particularly with fever, nausea, vomiting, or reduced food intake, injuries, reactive airway disease, inflammatory bowel disease and vigorous physical exercise, when blood glucose levels are persistently elevated (e.g., >14 mmol/L or 250 mg/dL) and in case of DKA symptoms (e.g., abdominal pain, fruity breath, lethargy) [115].

A retrospective analysis evaluating the clinical utility of blood ketone measurement in an adult population showed the need for cautious interpretation of blood ketone data alongside other biochemical parameters and suggest the development of improved diagnostic systems [116].

The latest advancements include portable, handheld devices that can measure β-hydroxybutyrate levels from capillary blood in just 10 s. This point-of-care technology allows for at-home or bedside testing, improving the management of ketone levels. Measuring blood ketones rather than traditional urine ketones has been shown to reduce hospitalizations for severe DKA [115]. However, adherence to ketone monitoring remains low. Effective sick-day management requires that patients frequently monitor both blood glucose and ketone levels to prevent metabolic decompensation [107].

Although portable ketone monitoring devices offer numerous advantages, such as convenience and accessibility, some limitations exist. These devices cannot detect the onset of DKA or track the progression of ketone levels without repeated point-of-care tests. Instead, they primarily confirm whether ketosis is already advancing, necessitating immediate medical intervention. To optimize their effectiveness, proactive sick-day guidelines must be provided to patients, outlining when and how to monitor ketone levels [117].

An international consensus has suggested that integrating continuous ketone monitoring with glucose monitoring into a single sensor could reduce the need for frequent separate tests. This combination could streamline the management of ketone levels and help to prevent severe DKA [117]. Teymourian et al. introduced the first continuous ketone monitoring system using a microneedle platform. This system employs electrochemical detection of β-hydroxybutyrate and can simultaneously measure glucose and lactate on the same microneedle array. The device integrates two distinct enzymatic biocatalytic systems, using dehydrogenase-oxidase chemistry, into a single sensing platform. This integration poses significant technical challenges, requiring meticulous design of both fabrication and detection protocols for each analyte. The development of this system marks a significant step forward in improving the quality of life for individuals with diabetes, offering enhanced monitoring capabilities that could help mitigate the risk of severe DKA while simplifying disease management [118].

## 6. Conclusions

Severe DKA in pediatric age is a multifaceted medical emergency that carries a potential risk of life-threatening complications. Early recognition, meticulous management, and prompt admission to PICU for high-risk children are critical in reducing complications and improving outcomes. To further mitigate the impact of this serious condition, comprehensive public awareness, education and screening campaigns are essential. These initiatives can empower families, caregivers, and healthcare providers to recognize early warning signs, seek prompt medical attention, and adhere to effective diabetes management strategies, also with the key role of new technologies. By combining clinical excellence with proactive community education, the burden and mortality associated with severe DKA in children can be significantly reduced.
